# Advanced Flow Cytometry Assays for Immune Monitoring of CAR-T Cell Applications

**DOI:** 10.3389/fimmu.2021.658314

**Published:** 2021-05-03

**Authors:** Ulrich Blache, Ronald Weiss, Andreas Boldt, Michael Kapinsky, André-René Blaudszun, Andrea Quaiser, Annabelle Pohl, Tewfik Miloud, Mégane Burgaud, Vladan Vucinic, Uwe Platzbecker, Ulrich Sack, Stephan Fricke, Ulrike Koehl

**Affiliations:** ^1^ Fraunhofer Institute for Cell Therapy and Immunology, Leipzig, Germany; ^2^ Institute of Clinical Immunology, Medical Faculty, University of Leipzig, Leipzig, Germany; ^3^ Beckman Coulter Life Sciences GmbH, Flow Cytometry Business Unit, Krefeld, Germany; ^4^ Beckman Coulter Life Sciences, Flow Cytometry R&D, Marseille, France; ^5^ Medical Faculty, Department of Hematology and Cell Therapy, University of Leipzig, Leipzig, Germany; ^6^ Institute for Cellular Therapeutics, Hannover Medical School, Hannover, Germany

**Keywords:** CAR-T, CD19, immune profiling, antibody, flow cytometry, staining technology

## Abstract

Adoptive immunotherapy using chimeric antigen receptor (CAR)-T cells has achieved successful remissions in refractory B-cell leukemia and B-cell lymphomas. In order to estimate both success and severe side effects of CAR-T cell therapies, longitudinal monitoring of the patient’s immune system including CAR-T cells is desirable to accompany clinical staging. To conduct research on the fate and immunological impact of infused CAR-T cells, we established standardized 13-colour/15-parameter flow cytometry assays that are suitable to characterize immune cell subpopulations in the peripheral blood during CAR-T cell treatment. The respective staining technology is based on pre-formulated dry antibody panels in a uniform format. Additionally, further antibodies of choice can be added to address specific clinical or research questions. We designed panels for the anti-CD19 CAR-T therapy and, as a proof of concept, we assessed a healthy individual and three B-cell lymphoma patients treated with anti-CD19 CAR-T cells. We analyzed the presence of anti-CD19 CAR-T cells as well as residual CD19+ B cells, the activation status of the T-cell compartment, the expression of co-stimulatory signaling molecules and cytotoxic agents such as perforin and granzyme B. In summary, this work introduces standardized and modular flow cytometry assays for CAR-T cell clinical research, which could also be adapted in the future as quality controls during the CAR-T cell manufacturing process.

## Introduction

Adoptive immunotherapy using immune effector cells engineered *ex vivo* to express chimeric antigen receptors (CARs) that mediate the lysis of cancer cells has become an innovative approach in cancer therapy (1). The CD19 antigen expressed on B cells has been the first clinically approved target of CAR-T cell immunotherapy. In comparison to conventional treatment approaches, anti-CD19 CAR-T cell treatment led to impressive remission rates in patients with precursor B-Cell Acute Lymphoblastic Leukemia (B-ALL), Diffuse Large B-Cell Lymphoma (DLBCL), Primary Mediastinal B-Cell Lymphoma (PMBCL) and Mantle Cell Lymphoma (MCL) (2–5). Spearheaded by this success, the potential of CAR-T cell therapy is currently being investigated in hundreds of clinical trials – the large majority in B cell malignancies (6). However, despite its great promise, CAR-T cell therapy bears several medical and economical challenges. Indeed, CAR-T cell therapy comes with severe, toxic and potentially life-threating adverse effects, which have been observed in a high proportion of patients (2–5, 7). These side effects are caused by cytokine secretion (e.g. IL-1, IL-6) due to immune cell and target interactions, which initiate cytokine release syndrome (CRS), macrophage activation syndrome (MAS) and neurotoxic symptoms (8–12). Beside body-imaging, monitoring of the patient’s immune system before and after CAR-T cell treatment and during further course of therapy could help to evaluate the success of the therapy and the status of the applied CAR-T cells. Furthermore, such patient monitoring could help to estimate the systemic immune response and might indicate the risk of immediate adverse effects or long-term complications. Focusing on B cell malignancies, at first the presence of both anti-CD19 CAR-T cells and remaining CD19+ B cells needs to be determined. Secondly, the fitness and activation status of the anti-CD19 CAR-T cells and the interacting patient´s immune cells might help to better understand the therapy course. Furthermore, the expression profiles of immune checkpoint molecules, co-inhibitory and co-stimulatory receptors, and differentiation markers of T lymphocytes are of high interest, because ligand interactions with these molecules can modulate endogenous T cell and CAR-T cell efficacy.

Methods to assess these parameters must be highly standardized, robust, and transferable. Multiparametric immunophenotyping by flow cytometry allows the fast, comprehensive and mostly antibody-based routine analysis of progenitor and immune cells in the peripheral blood, bone marrow or cerebrospinal fluid in healthy people and in patients with leukemia and lymphoma (13–15). However, flow cytometry is prone to variability due to individual operator handling, reagent/antibody issues and data analysis (16). Pre-formulated dry antibody panels that are expert-designed and produced under standardized conditions can contribute to minimizing these sources of human variability and technical issues. DURA Innovations (Beckman Coulter Life Sciences, Brea CA/USA) is a research technology that supports the standardization of flow cytometry through a non-lyophilized layer of pre-formulated, dry antibodies at the bottom of a ready-to-use test tube. In addition, tandem dye conjugates used in the DURA Innovations format ensure consistency without the need for refinement of spillover correction regardless of how experienced an operator might be. These pre-formulated antibody panels can be complemented by the addition of further antibodies in liquid format to match specific requirements related to CAR-T cell therapy, research applications or the quality control (QC) of CAR-T cell production.

In this research study, we apply a comprehensive set of standardized flow cytometry assays to phenotype the cellular immune system. As a proof of concept, we examine the immune cells present in blood samples of three CAR-T cell treated patients suffering from B cell lymphoma in comparison to respective controls. We show that the flow cytometry assays are suitable to track the CAR-T cells, to assess the fitness of the T cell compartment and to measure further accompanying factors in the blood of the patient’s immune system. We believe that the novel 13-colour/15-parameter flow cytometry assays could help to monitor the course of CAR-T immunotherapy in the future. Furthermore, the applied assays might also serve as a novel QC tool to monitor the manufacturing of CAR-T cell products.

## Materials and Methods

### Human Blood Samples

EDTA-anticoagulated (Sarstedt, Nümbrecht, Germany) or heparinized whole blood was obtained from a healthy donor and from three patients (DLBCL, PMBCL, transformed Follicular Lymphoma) treated with anti-CD19 CAR-T cells (Axicabtagene ciloleucel or Tisagenlecleucel), which gave informed consent. Ethical approval was given by the local Ethic committee at the University of Leipzig (351/17-ek).

### Instrument Settings

In our study, the *Minimum Information about a Flow Cytometry Experiment* (MiFlowCyt) (17) were considered if reasonable. A DxFLEX flow cytometer equipped with 3 Lasers (405/488/638nm), 13 fluorescence detectors and a standard filter configuration (Beckman Coulter Life Sciences, Brea CA/USA) was used in this study. Setup, calibration and quality control procedures (QC) were conducted according to the manufacturer’s instructions. In brief, to set up the DxFLEX instrument, Daily QC beads (Beckman Coulter Life Sciences, Brea CA/USA) were used. For compensation, either single color-stained lysed whole blood samples or single color-stained VersaComp antibody capturing beads (Beckman Coulter Life Sciences, Brea CA/USA) were used and an automatic compensation was performed according to the manufacturer’s instructions. The filter specifications, the fluorochrome labels used and detectors are shown in **Table 1**.

**Table 1:** Filter specifications, fluorochrome labels used and detectors applied in this study.

Excitation Filter	Violet Laser 405 nm	Blue Laser 488 nm	Red Laser 638 nm
**450/45 BP**	Pacific Blue, SNv428, ViaKrome 405		
**525/40 BP**	Krome Orange	FITC	
**585/42 BP**		PE	
**610/20 BP**	BV605, Superbright 600	ECD	
**660/10 BP**	BV650		APC
**690/50 BP**		PC5.5	
**712/25 BP**			APC-AF700, AF700
**780/60 BP**	BV785	PC7	APC-AF750, AF750

### Leukocyte Counting and Viability

Leukocytes counting and cell viability were performed using the ready-to-use DURAClone IM-count tube containing anti-CD45-FITC, 7-AAD and reference beads (Beckman Coulter Life Sciences, Brea CA/USA). 100 µl of EDTA-anti-coagulated peripheral blood was added to the DURAClone IM count tube (Beckman Coulter Life Sciences), followed by 6-8 sec of mixing and 15 min of incubation at room temperature (RT) in the dark. Next, 2 ml of Versalyse (Beckman Coulter Life Sciences) were added for red blood cell lysis, followed by mixing, 10 min incubation at RT in the dark and data acquisition on the flow cytometer. The obtained CD45+ leukocyte absolute count is used to estimate cellular concentrations for CD45+ subpopulations in all tubes with a CD45 staining.

### Surface Marker Immunostaining

The antibody panels TCR, RE ALB, CAR-T1, CAR-T2, NK1, NK2 were used to stain the EDTA blood samples. The CAR-T1, CAR-T2, NK1 and NK2 were custom design panels in dry DURA Innovations format and are not available as off-the-shelf products.

After the addition of further liquid drop-in antibodies to the dry antibody panels, 100 µl of EDTA-anti-coagulated whole blood was added, followed by mixing and 15 min incubation at RT in the dark. Red blood cells were lysed by adding 2 ml of VersaLyse, followed by mixing and incubation for 10 minutes in the dark. Lysed cells were centrifuged at 200 g for 5 min, the supernatant was discarded and cells were washed with 3 ml of phosphate buffered saline (PBS, 10x Gibco, 70011-036). After centrifugation (200 g, 5 min), the supernatant was discarded and cells were resuspended in 0.5 ml of PBS, containing 0.1% formaldehyde prior to acquisition on the flow cytometer.

### Intracellular Immunostaining

For the antibody panel CAR-T3 (intracellular proteins) 50 µl of heparinized blood was stimulated using the dry DURActive 1 kit containing phorbol 12-myristate 13-acetate (PMA), Ionomycin and Brefeldin A (Beckman Coulter Life Sciences) for 3 h at 37°C, or control stimulated using PBS. Activated samples were prepared using the PerFix-nc buffer system (please see Beckman Coulter’s detailed instructions for the use of PerFix-nc, product ref. B31167). The activated material was fixed with buffer R1 for 15 min. In the meantime, 300 µl of buffer R2 was added to the dry CAR-T3 antibody panel and the additional liquid drop-in antibodies before adding the fixed sample to the CAR-T3 tube. Red blood cell lysis occurs synchronously to permeabilization and staining during incubation for 30 min at RT in the dark. Stained samples were washed with 3 ml of buffer R3, centrifuged (200 g, 5 min) and the cell pellet was resuspended in 0.5 ml buffer R3 prior to acquisition.

### Antibodies

The antibodies and dyes used in this study are shown in **Table 2**.

**Table 2:** Antibodies and dyes used in this study.

Antibody	Clone	Fluorochrome(s)	Volume per test	Product ref.	Vendor
7-AAD	n.a.	n.a.	n.a.	C00162 (DURAClone IM Count)	Beckman Coulter
aCD19 CAR Biotin	n.a.	n.a.	5 µl	130-115-965	Miltenyi Biotec
anti Biotin	REA746	PE	5 µl	130-110-951	
CD3	UCHT-1	APC-AF750, AF750	n.a.	B53340 (DURAClone IM TCR), DURAClone custom	Beckman Coulter
CD4	13B8.2	APC	n.a.		Beckman Coulter
CD8	B9.11	Krome Orange, AF700	n.a,		Beckman Coulter
CD10	ALB1	PC5.5	n.a.	C00163 (DURAClone RE ALB)	Beckman Coulter
CD15	80H5	Pacific Blue	10 µl	B49218	Beckman Coulter
CD16	3G8	FITC	n.a.	DURAClone custom	Beckman Coulter
CD19	J3-119	PC7	n.a.	C00163 (DURAClone RE ALB)	Beckman Coulter
CD20	B9.E9	APC-AF750	n.a.		Beckman Coulter
CD22	SJ10.1H11	APC	10 µl	A60791	Beckman Coulter
CD24	ALB9	APC	10 µl	A87785	Beckman Coulter
CD25	B1.49.9	SNv428	n.a.	DURAClone custom	Beckman Coulter
CD27	1A4CD27	PC5.5	10 µl	B21444	Beckman Coulter
CD28	CD28.2	BV650	5 µl	302946	Biolegend
CD31	WM59	BV605	5 µl	303121	Biolegend
CD34	581	ECD	n.a.	C00163 (DURAClone RE ALB)	Beckman Coulter
CD38	LS198-4-3	APC-AF700	n.a.		Beckman Coulter
CD45	J33	Krome Orange	n.a.	B53340 (DURAClone IM TCR), C00163 (DURAClone RE ALB), DURAClone custom	Beckman Coulter
CD45RA	HI100	BV785	5 µl	304140	Biolegend
CD56	N901	APC-AF750	n.a.	DURAClone custom	Beckman Coulter
CD56	5.1H11	BV785	5 µl	362550	Biolegend
CD57	NC1	FITC	n.a.	DURAClone custom	Beckman Coulter
CD58	AICD58	FITC	n.a.	C00163 (DURAClone RE ALB)	Beckman Coulter
CD69	TPI.55.3	PC7	n.a.	DURAClone custom	Beckman Coulter
CD95	DX2	BV650	5 ul	305642	Biolegend
CD127	R34.34	FITC	n.a.	DURAClone custom	Beckman Coulter
CD134 (OX40)	Ber-ACT35	BV650	5 µl	563658	BD Biosciences
CD137 (4-1BB)	4B4-1	ECD	n.a.	DURAClone custom	Beckman Coulter
CD155 (PVR)	SKII.4	BV650	5 µl	748275	BD Biosciences
CD197 (CCR7)	GO43H7	BV605	5 µl	353224	Biolegend
CD223 (LAG-3)	11C3C65	ECD	n.a.	DURAClone custom	Beckman Coulter
CD226	11A8	BV785	5 µl	338322	Biolegend
CD274 (PD-L1)	PD-L1	PC7	n.a.	DURAClone custom	Beckman Coulter
CD278 (ICOS)	C398.4A	BV785	5 µl	313534	Biolegend
CD279 (PD-1)	PD1.3	PC5.5	n.a.	DURAClone custom	Beckman Coulter
CD314 (NKG2D)	ON72	APC	n.a.		Beckman Coulter
CD335 (NKp46)	BAB281	PE	n.a.		Beckman Coulter
CD366 (TIM-3)	F38-2E2	SNv428	n.a.		Beckman Coulter
HLA-DR	Immu357	ECD, PC5.5	n.a.		Beckman Coulter
Granzyme B	GB11	ECD	n.a.		Beckman Coulter
INFγ	45.15	FITC	n.a.		Beckman Coulter
IL-2	MQ1-17H12	PC7	n.a.		Beckman Coulter
Perforin	dG9	PC5.5	n.a.		Beckman Coulter
TCRαβ	IP26A	PE	n.a.	B53340 (DURAClone IM TCR)	Beckman Coulter
TCRγδ	IMMU510	FITC	n.a.		Beckman Coulter
TCRVδ1	R9.12	PC7	n.a.		Beckman Coulter
TCRVδ2	IMMU389	Pacific Blue	n.a.		Beckman Coulter
TIGIT	A15153G	BV605	5 µl	372712	Biolegend
TNFα	IPM2	AF700	n.a.	DURAClone custom	Beckman Coulter
ViaKrome 405	n.a.	n.a.	2.5 µl (reconstituted)	C36614	Beckman Coulter
VISTA	B7H5DS8	Superbright600	5 µl	No longer available	Thermo Fisher

### Data Analysis

All dot plots were generated using Kaluza 2.1 analysis software (Beckman Coulter Life Sciences).

## Results

### Pre-Formulated Dry Antibody Assays for Flow Cytometry

**Figure 1:**
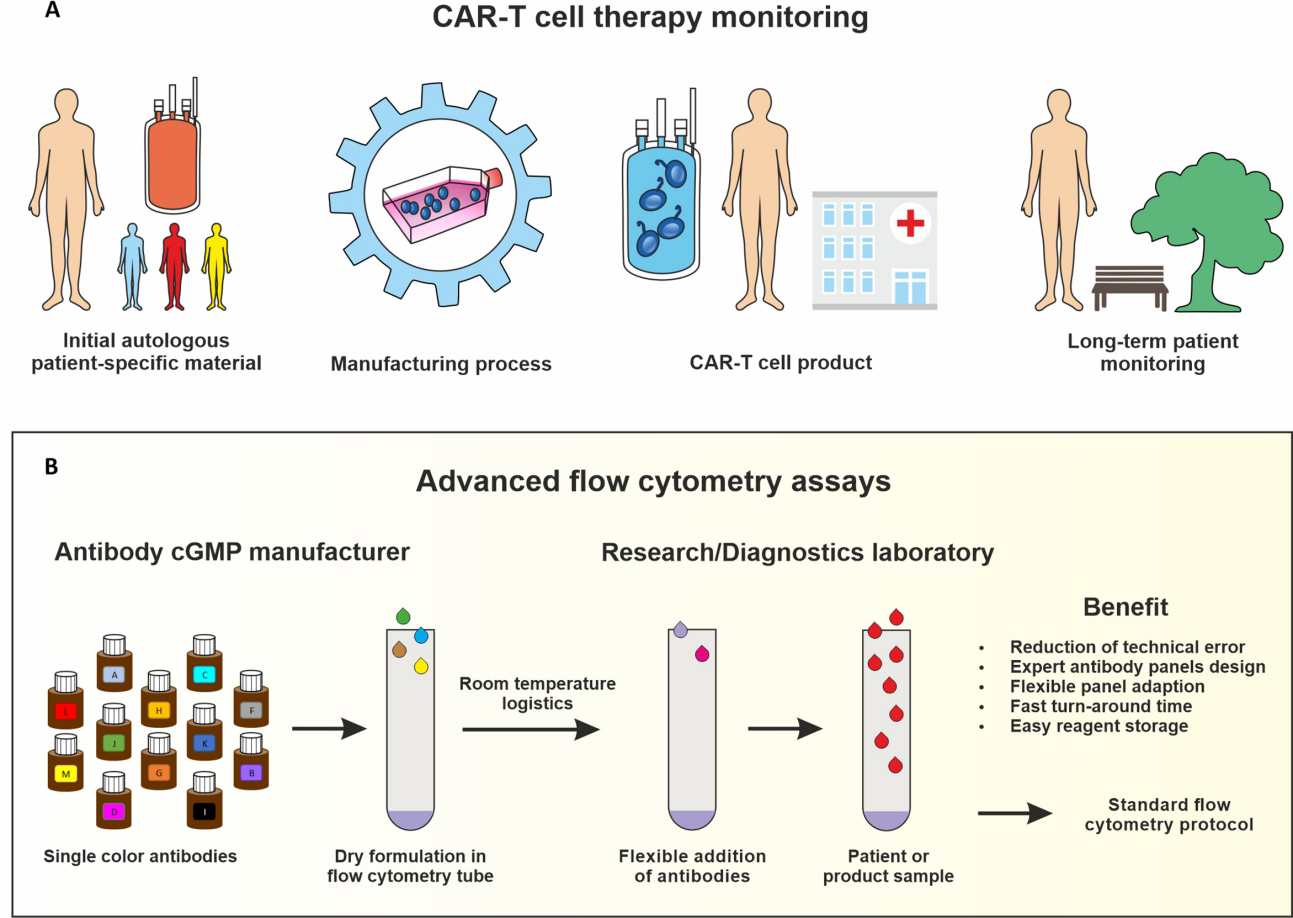
IImmune monitoring assays for CAR-T cell production and therapy. Key steps of the CAR-T cell therapy that should be monitored include analyzing the starting leukapheresis patient material, the manufacturing process, the CAR-T cell product release and the long-term patient follow-up **(A)**. Advanced flow cytometry assays based on dry uniform DURAClone antibody panels for standardization which can be complemented with antibodies in liquid formulation for specific purposes **(B)**.

**Figure 2:**
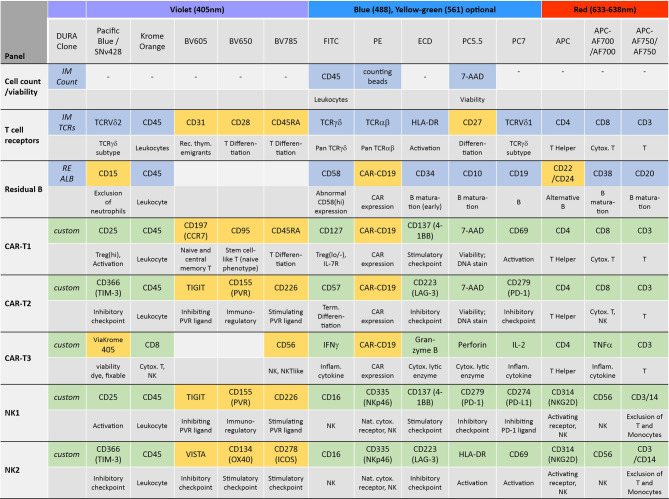
Antibody panel design. The antibody composition of the designed panels is shown together with the biological implication of each assessed antigen (grey) and the used fluorochromes and lasers. Antibody panels compose of dry antibodies that are off-the-shelf DURAClone products (blue) or custom-made DURAClone products (green). These dry antibody-based panels are complemented with further antibodies in liquid formulation (orange).

In order to establish a standardized flow cytometry method for CAR-T monitoring, we developed assays that are based on pre-formulated dry antibody panels usefull at all steps of CAR-T cell therapy (**Figure 1**). These flow cytometry assays are produced so that the antibodies are present in dried form at the bottom of the reagent tube and the (blood) sample can be directly added to the pre-formulated antibody mixtures. In our study we established assays consisting of comprehensive immune cell antibody backbone panels, which we complemented with accompanying antibodies to best fit our purpose of phenotyping the cellular immune systems of CAR-treated patients. We have carefully configured all our antibody panels so that antigen density and fluorochrome brightness are reciprocally matched and that spill over situations are minimized or not critical. The exact antibody composition of all established panels together with the fluorochromes used is shown in **Figure 2**.

### Leukocyte Viability and Detection of CD19+ B Cells

**Figure 3:**
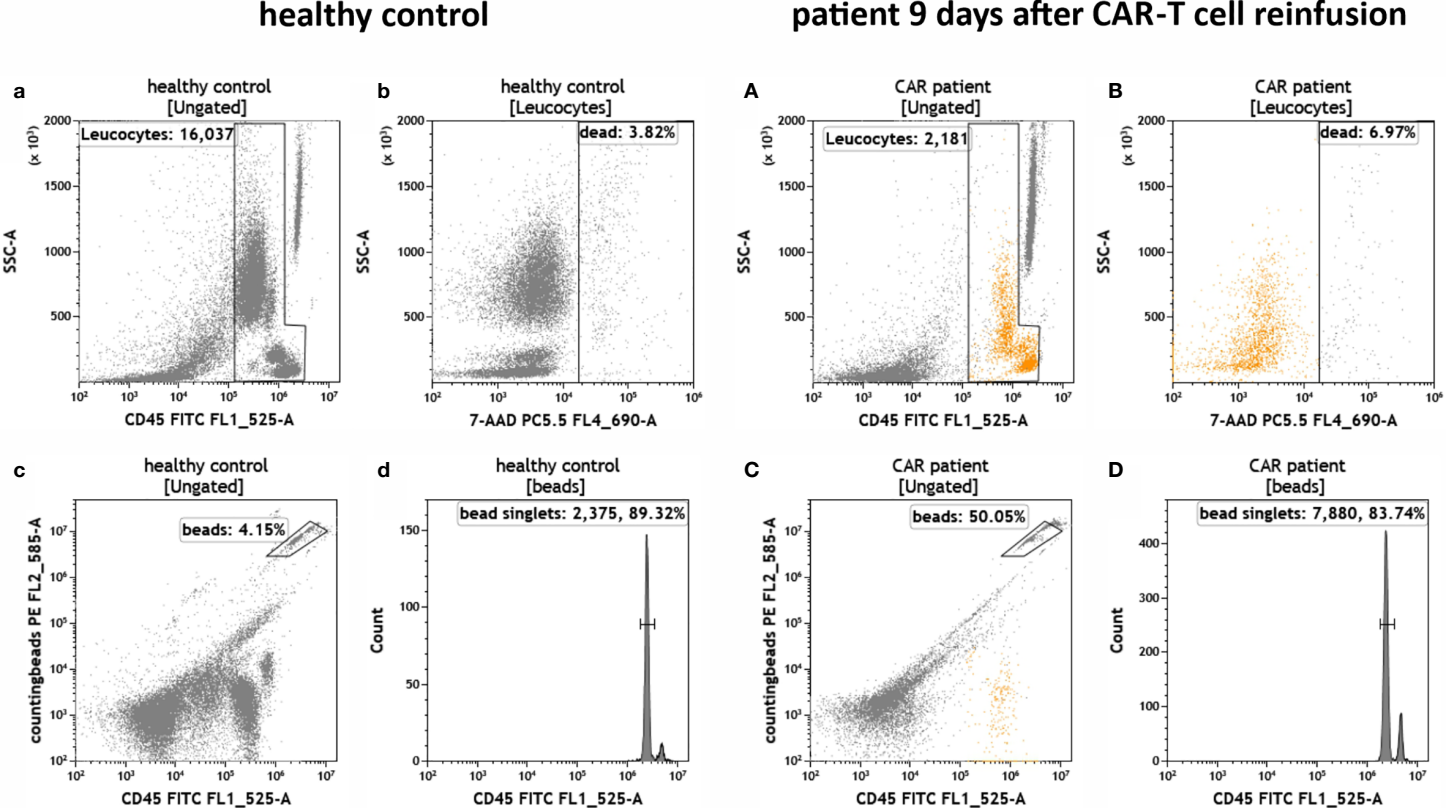
Total number and viability of leukocytes in the peripheral blood of a healthy individual and a patient treated with anti-CD19 CAR-T cells. Flow cytometric identification of leukocytes **(a, A)** and cell counting by bead measurement **(c, C)** in both healthy control **(a–d)** and a patient 9 days after CAR-T cell reinfusion **(A–D)**. Dead cells **(b, B)** and bead doublets **(d, D)** were excluded.

**Figure 4:**
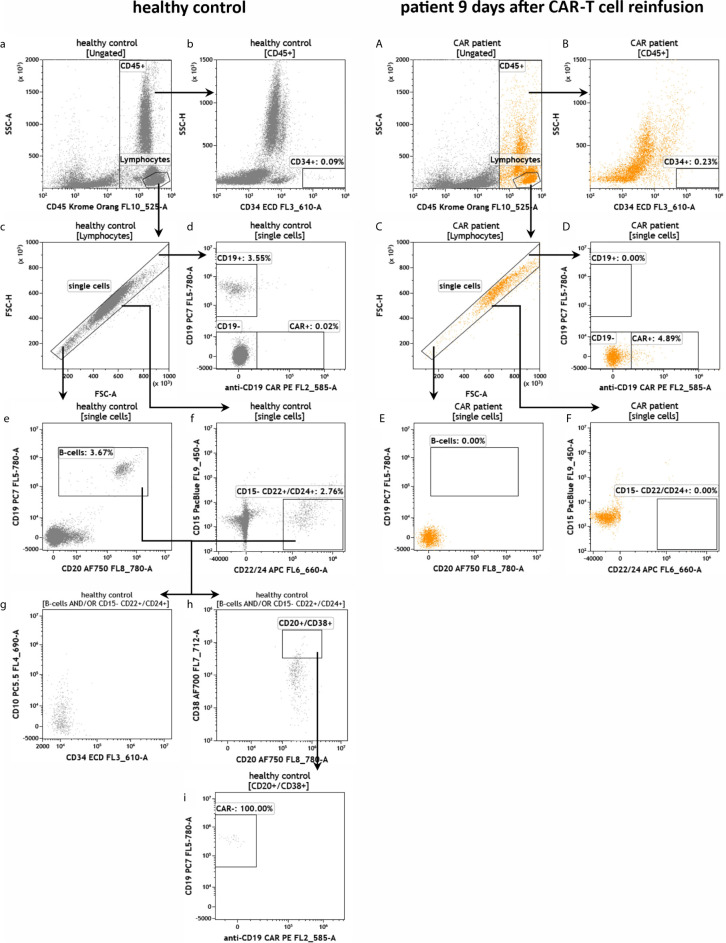
Detection of CD19+ B cells in the peripheral blood of a healthy individual and a patient treated with anti-CD19 CAR-T cells. Flow cytometric gating strategy (illustrated by arrows) and phenotyping of B cells in healthy control **(a–i)** in comparison to residual B cells in a patient 9 days after anti-CD19 CAR-T cell reinfusion **(A–F)**. After lymphocyte identification **(a, A)** CD34+ progenitor cells were detected **(b, B)** and exclusion of doublets was performed **(c, C)**. The presence of potential CAR+ CD19 B cells was evaluated **(d, D)**. B cells and residual B cells were identified by gating CD19, CD20 and CD22/24 **(d, D–f, F)**. If present, B-cells can be further characterized by CD10, CD34, CD38 and anti-CD19 CAR **(g-i)**.

To test the established assays, we used peripheral blood samples collected from a healthy donor and from a patient suffering from DLBCL treated with CD19-directed CAR-T cells (Axicabtagene ciloleucel). The blood sample from the DLBCL patient was obtained 9 days post-infusion when CAR-T cells typically peak in the peripheral blood (3, 5, 7, 18, 19). The blood samples were added to the test tubes, processed and measured on the flow cytometer as described in the experimental section. First, we determined the number of leukocytes in the blood samples and their viability (IM-count tube) (**Figure 3**). Overall, we found that the anti-CD19 CAR patient had much fewer leukocytes than the healthy control due to prior lymphodepleting therapy (**Figures 3a, A**) and that leukocyte viability was high (>93%) in both processed samples (**Figures 3b, B**). Next, we examined the CD19+ B cells in both samples (RE ALB tube) (**Figure 4**). Among the lymphocytes of the healthy individual we detected 3.55% CD19+ B cells (**Figures 4a–d**), which were largely also positive for the B cell maturation markers CD20 and CD22/24 (**Figures 4e–f**). These B cells were mostly negative for CD10, CD34 and CD38 (**Figures 4g–i**). In contrast, we could not find any residual CD19+ B cells in the anti CD19 CAR-T cell treated patient (**Figures 4A–F**). Furthermore, we could also not detect any anti-CD19 CAR+ CD19+ cells in the patient sample (**Figure 4D**).

### Assessment of the T Cell Compartment and Anti-CD19 CAR-T Cell Fitness

**Figure 5:**
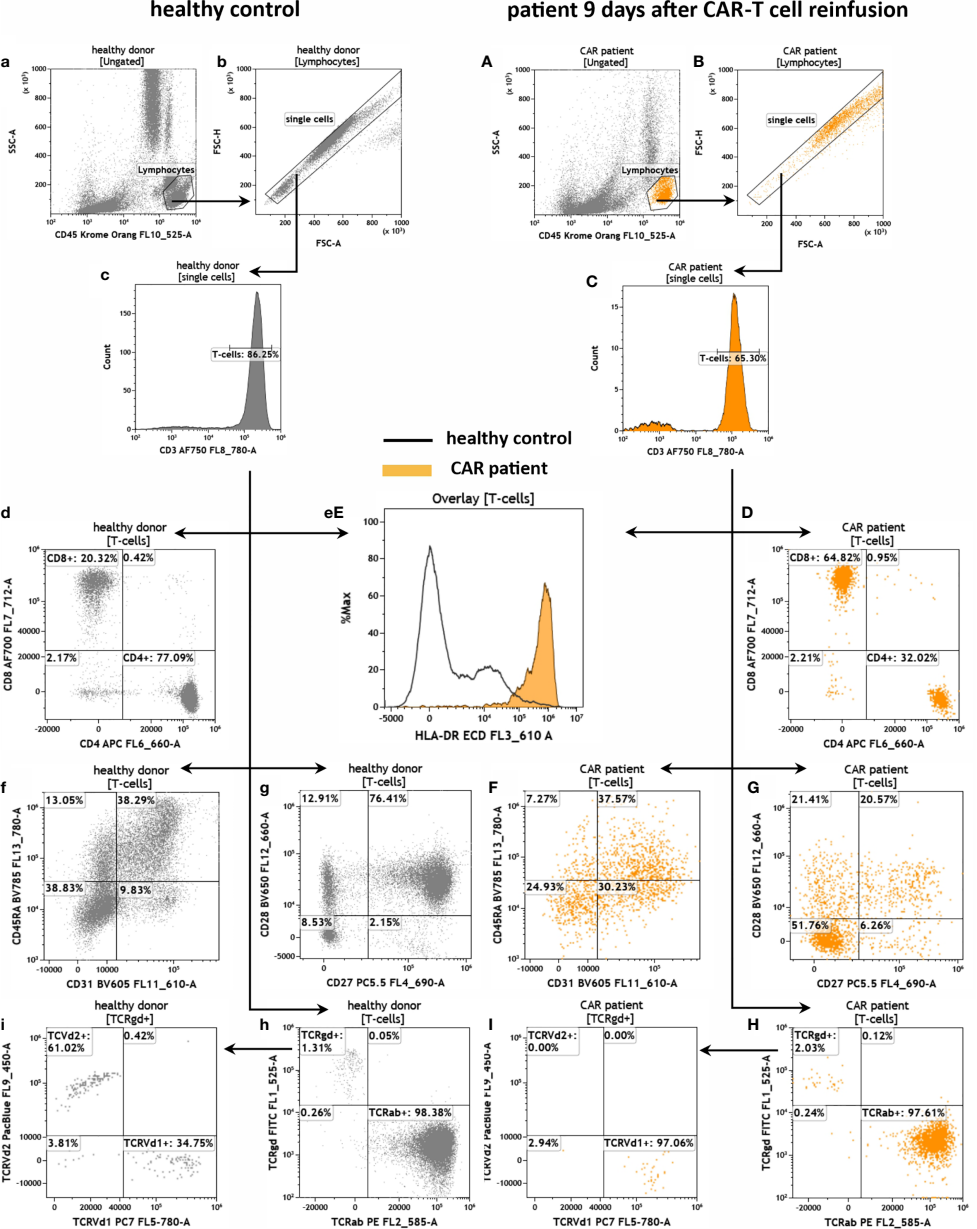
Assessment of T cells in the peripheral blood of a healthy individual and a patient treated with anti-CD19 CAR-T cells by the panel TCR. Flow cytometric gating strategy (illustrated by arrows) and phenotyping of T cells in healthy control **(a–h)** in comparison to a patient 9 days after CAR-T cell reinfusion **(A–H)**. After lymphocyte gating **(a, A)** and exclusion of doublets **(b, B)** CD3+ T cells were identified **(c, C)**. T cells were further discriminated in CD4+ and CD8+ T cells **(d, D)**, CD31+CD45RA+ recent thymic emigrants **(f, F)** and naïve/memory (CD27+/CD28+/-) as well as effector-memory (CD27-/CD28-) cells **(g, G)**. The expression of HLA-DR on T cells **(eE)** was used as a marker for general T cell activation. Furthermore, the numbers of alpha/beta and gamma/delta T cells **(h, H)** as well as Vd1+ and Vd2+ gamma/delta T cells were determined **(i, I)**.

To investigate the T cell compartment we used a T cell receptor (TCR) antibody panel (**Figure 5**). First, we gated for CD3+ and found that 86.25% and 65.30% of the lymphocytes were T cells in the healthy and CAR treated patient sample, respectively (**Figures 5a–c, 5A–C**). Afterward, we discriminated the CD3+ population in CD4+ and CD8+ T cells and observed a CD4/CD8 ratio of 3.8 (77.09%/20.32%) in the healthy individual (**Figure 5d**). Vice versa, in the CAR treated patient we found an inversed CD4/CD8 ratio of 0.5 (32.02%/64.82%) as can be seen in **Figure 5D**. Furthermore, the T cell activation status was much higher in the CAR treated patient than in the healthy individual as measured by HLA-DR expression (**Figure 5eE**). We found a similar percentage of 38% CD31+CD45RA+ recent thymic emigrants in both samples (**Figures 5f, F**). In the CAR treated patient 20.57% of all T cells were CD27+/CD28+ memory T cells, whereas in the healthy donor 76.41% of all T cells were CD27+/CD28+ (**Figures 5g, G**). Furthermore, we also determined the percentage of alpha/beta T cells and Vd1+ or Vd2+ gamma/delta T cells (**Figures 5h, H, i, I**) and we identified the lack of Vd2 gamma/delta T cells (TCVd2) in the CAR treated patient to be the main difference between both samples (**Figures 5i, I**).

**Figure 6:**
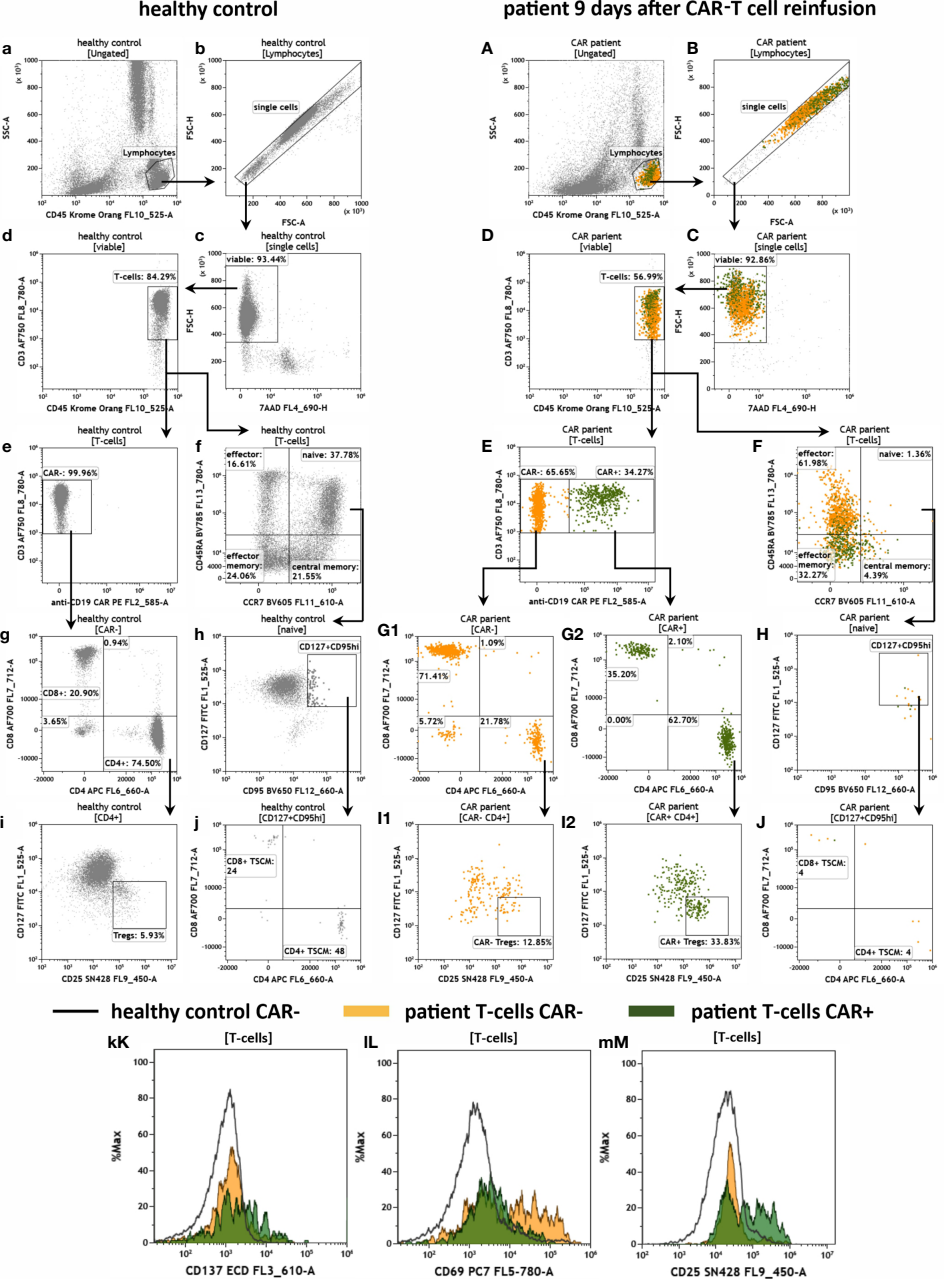
Characterization of anti-CD19 CAR-T cells in the peripheral blood of a patient treated with CAR-T cells by the panel CAR-T1. Flow cytometric gating strategy (illustrated by arrows) and phenotyping of anti-CD19 CAR-T cells in a patient 9 days after CAR-T cell reinfusion **(A–M)** in comparison to a healthy donor **(a–m)**: After lymphocyte gating **(a, A)** and exclusion of doublets **(b, B)** viable **(c, C)** T cells **(d, D)** were identified. The T cells were divided in anti-CD19 CAR- and CAR+ cells **(e, E)**. CAR- and CAR+ T cells were further divided in CD4+ and CD8+ cells **(g, G1** and **G2)**, followed by identification of CD127dimCD25+Tregs in the CD4+ T cell populations **(i, I1** and **I2)**. CAR-T cells and normal T cells maturation stages were assessed by CD45RA and CCR7 expression **(f, F)**. In the naïve compartment, CD127+CD95+ cells are assessed for identification of a stem cell-like T cell memory type **(h, H)** including expression of CD4 and CD8 **(j, J)**. Expression of the checkpoint CD137 and the activation markers CD69 and CD25 is compared by overlay plots between T cells from the healthy donor and patient CAR+ and CAR- T cells. **(kK–mM)**. The healthy control did not have anti-CD19 CAR-T cells. Color code: In the CAR-T cell treated patient, events are assigned to either anti-CD19 CAR- (orange) or CAR+ (green) T cells.

Next, we aimed to characterize the anti-CD19 CAR-T cells in the peripheral patient blood. To this end, we applied the antibody panel CAR-T1 (**Figure 6**). As observed before in **Figure 5**, the healthy donor had much more leukocytes and the cell viability was high in both samples (**Figures 6a–c, A–C**). We also confirmed that the percentage of CD45+/CD3+ T cells in the lymphocyte population was higher in the healthy than in the CAR treated individual (**Figures 6d, D**). When analyzing the CD3+ cells in more detail, we detected no anti-CD19 CAR-T cells in the healthy donor, and therefore no false-positive results of the anti-CD19 CAR-T staining (**Figure 6e**). We further confirmed the high CD4/CD8 ratio of 3.6 (74.50%/20.90%) in the healthy individual (**Figure 6g**). In the CAR treated patient 34.27% of all CD45+/CD3+ T cells were positive for the anti-CD19 CAR receptor (**Figure 6E**). Interestingly, in this anti-CD19 CAR+ T cell population we found a CD4/CD8 ratio of 1.8 (62.70%/35.20%; **Figure 6G2**), whereas in the anti-CD19 CAR- population the CD4/CD8 ratio was only 0.3 (21.78%/71.41%; **Figure 6G1**), which is consistent with our previous data (see **Figure 5**). The CAR-T1 panel is also designed to stain for markers of different effector and memory T cells subpopulations (CCR7, CD45RA, CD127, CD95). Gating for these markers revealed very low percentages of naïve and central memory cells in the patient T cells in comparison to the healthy donor (**Figures 6f, F, h, H**). Next, we identified CD127dimCD25+ T regulatory cells (Tregs) in the CD4+ T cell populations and found Tregs in both samples with Tregs percentages being the highest in the CAR+ patient T cells (**Figures 6i, I1, I2**). Using overlay plots we visualized that expression of the checkpoint molecule CD137 is similar between the CAR patient and healthy donor (**Figure 6kK**), whereas the activation markers CD69 and CD25 were higher in CAR- and CAR+ cells, respectively (**Figures 6lL, mM**).

**Figure 7:**
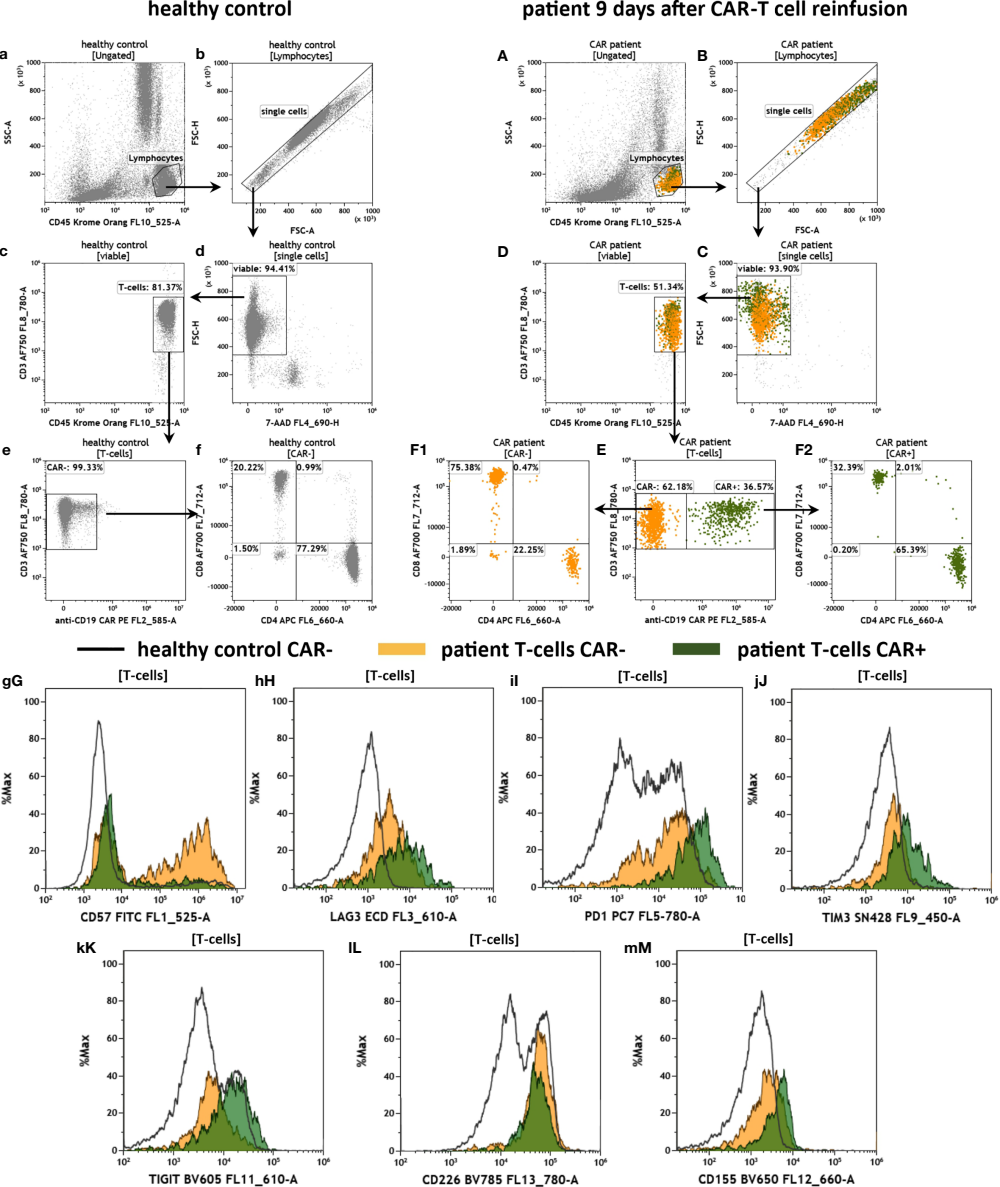
Fitness of anti-CD19 CAR-T cells in the peripheral blood of a patient treated with CAR-T cells by the panel CAR-T2. Flow cytometric gating strategy (illustrated by arrows) and phenotyping of anti CD19 CAR-T cells in a patient 9 days after CAR-T cell reinfusion **(A–M)** in comparison to a healthy control individual **(a–m)**: After lymphocyte gating **(a, A)** and exclusion of doublets **(b, B)** viable **(c, C)** T cells **(d, D)** were identified. The T cells were divided in anti-CD19 CAR- and CAR+ cells **(e, E)**. CAR- and CAR+ T cells were further divided in CD4+ and CD8+ cells **(f, F1**, **F2)**. T cell expression of the terminal differentiation marker CD57 **(gG)**, checkpoints LAG3 **(hH)**, PD-1 **(iI)**, TIM3 **(jJ)**, and the immune modulating TIGIT **(kK)**, CD226 **(lL)** and CD155 **(mM)** were compared by overlay plots between T cells from the healthy donor and patients CAR- and CAR+ T cells. The healthy control did not have anti CD19 CAR-T cells. Color code: In the CAR-T cell treated patient, events are assigned to either anti-CD19 CAR- (orange) or CAR+ (green) T cells.

The purpose of the CAR-T2 panel is to assess the CAR-T cell fitness through staining of T cell exhaustion and immune checkpoint molecules (**Figure 7**). Therefore, the panel is similar in design to the panel CAR-T1 and anti-CD3, -CD4, -CD8, -CD45 and anti-CD19 CAR antibodies are present in the panel CAR-T2 as well (see **Figure 2**). Consequently, the data shown in **Figures 7a–f** and **7A–F** are reproducing the results from **Figure 6**. A key marker of the panel CAR-T2 is CD57, which indicates the terminal differentiation and exhaustion of T cells. For this exhaustion marker we observed a stronger expression in the CAR- T cells of the CAR treated patient in comparison to the CAR+ T cells, and also for the T cells of the healthy donor (**Figure 7gG**). Furthermore, we measured several inhibitory immune checkpoint molecules (LAG3, PD-1, TIM3) as well as the TIGIT/CD226/CD155 immune checkpoint signaling axis (**Figures 7hH–mM**). For all assessed checkpoint molecules, we found a trend toward higher expression in the T cells of the CAR patient compared to the healthy individual, and a tendency of higher expression in the CAR+ than in the CAR- patient T cells.

**Figure 8:**
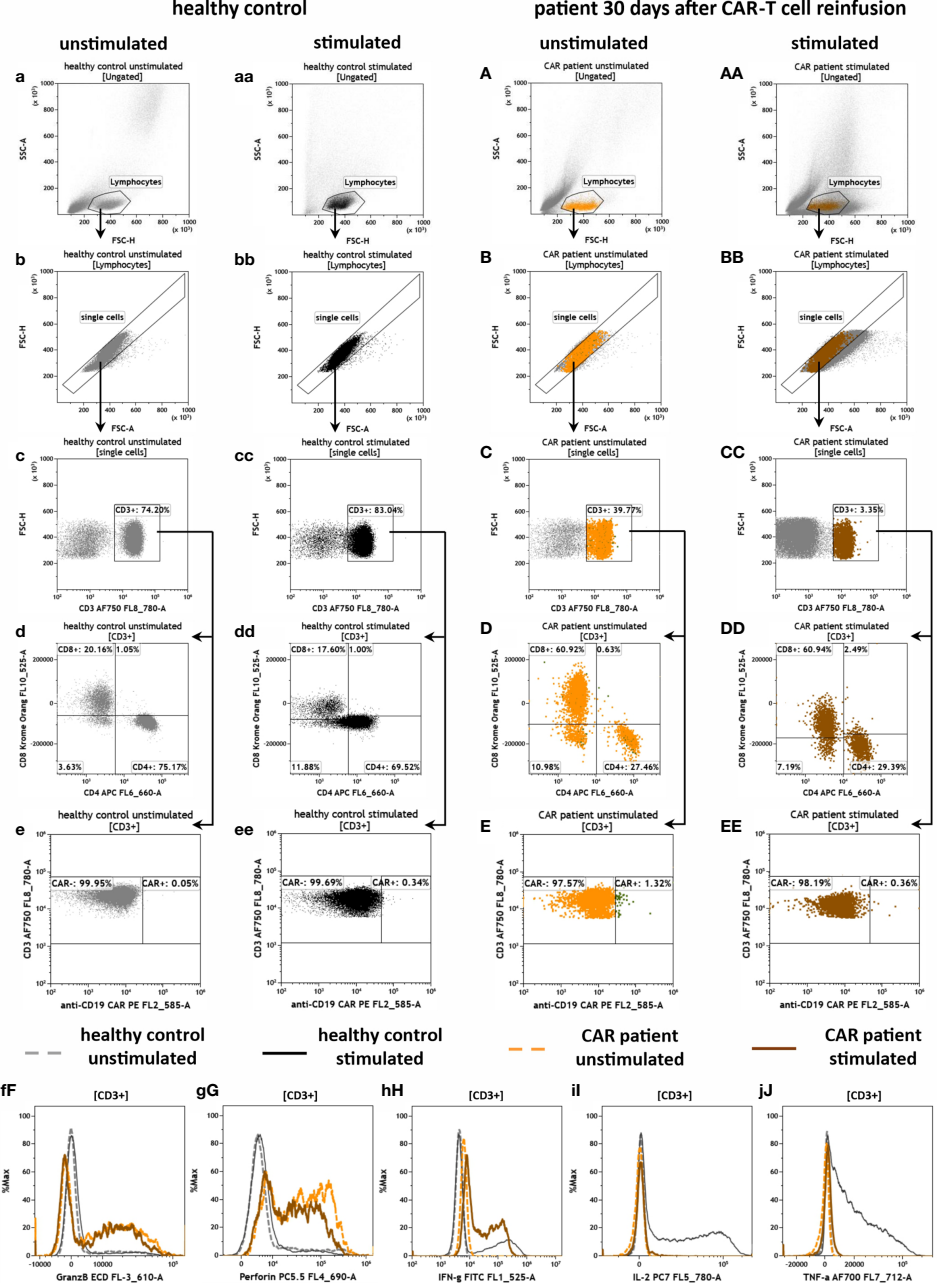
Effector potential of anti-CD19 CAR-T cells in the peripheral blood of a patient treated with CAR-T cells by the panel CAR-T3. Flow cytometric gating strategy (illustrated by arrows) and phenotyping of extra- and intracellular staining of anti-CD19 CAR-T cells in a patient 30 days after CAR-T cell reinfusion **(A–J)** in comparison to a healthy donor **(a–j)**, in both unstimulated cells **(a–j** and **A–J)** or cells that were stimulated with PMA/Ionomycin **(aa–j, AA–J)**: After lymphocyte gating **(a–AA)** and exclusion of doublets **(b–BB)**, T cells **(c–CC)** were identified and further divided in CD4+ and CD8+ T cells **(d–DD)** as well as CAR- and CAR+ T cells **(e–EE)**. The intracellular expression of granzyme B **(fF)**, perforin **(gG)**, IFN-gamma **(hH)**, IL-2 **(iI)** and TNF-α **(jJ)** were compared by overlay plots between unstimulated and stimulated T cells from the healthy donor and the anti-CD19 CAR-T cell treated patient. The healthy control did not have CAR-T cells. Color code: dashed gray line = unstimulated healthy donor; black = stimulated healthy donor; dashed orange = unstimulated anti CD19 CAR patient, brown = stimulated anti CD19 CAR patient.

To evaluate the effector function of CAR-T cells by measuring intracellular markers we developed the antibody panel CAR-T3 (**Figure 8**). Therefore, blood of the CAR patient 30 days after CAR-T cell re-infusion and blood of the healthy donor was stimulated for 3 h at 37°C in DURActive 1 tubes. The backbone of CAR-T3 was the same as for the other CAR-T tubes. After gating of lymphocytes (**Figures 8a, aa, A, AA**) and single cells (**Figures 8b, bb, B, BB**) T cells were identified (**Figures 8c, cc, C, CC**). As for CAR-T1 (**Figure 6**) and CAR-T2 (**Figure 7**) T cells were lower in the CAR patient compared to the healthy donor (**Figures 8c, cc, C, CC**). Furthermore, the CD4/CD8 ratio was 3.73 (75.17%/20.16%) in the unstimulated healthy control and 3.95 (69.52%/17.60%) in the stimulated one (**Figures 8d, dd**) while for the CAR patient the ratios were 0.45 (27.46%/60.92%) and 0.49 (29.39%/60.94%) (**Figures 8D, DD**), respectively. As expected the CAR-T cell content in the blood of the CAR patient was much lower (**Figures 8E, EE**) than after 9 days of re-infusion (**Figures 6** and **7**). Stimulation of the cells does not alter the amount of intracellular granzyme B (**Figure 8fF**) and perforin (**Figure 8gG**). However, levels of granzyme B (**Figure 8fF**) and perforin (**Figure 8gG**) of the CAR patient sample were higher compared to the healthy donor. For IFN-γ the stimulation leads to an intracellular enhancement in the CAR patient and the healthy donor (**Figure 8hH**) while for IL-2 (**Figure 8iI**) and TNF-α (**Figure 8jJ**) an enhancement was only observed in the healthy control.

**Figure 9:**
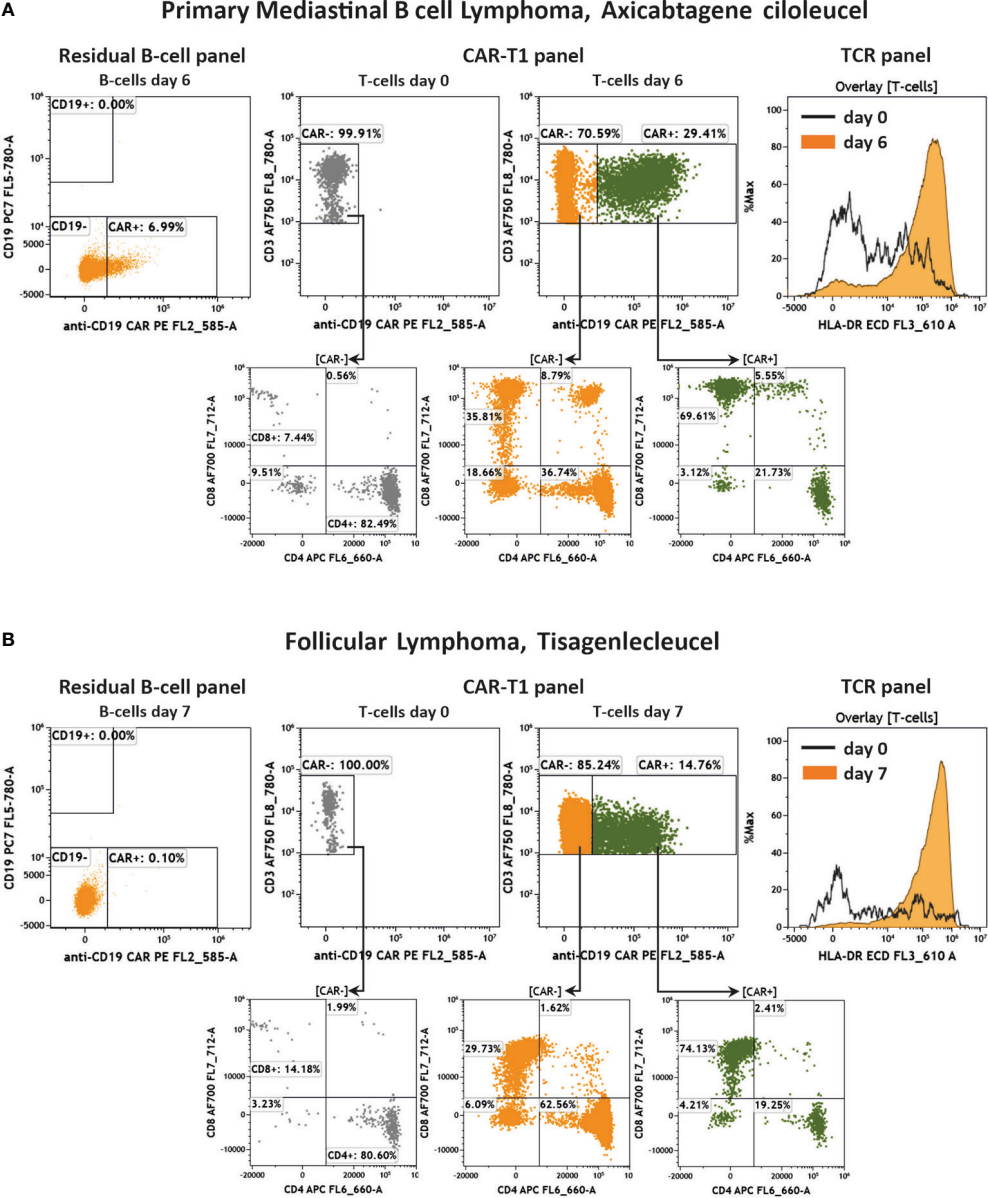
Key results of two additional anti-CD19 CAR-T cell treated patients. Flow cytometric analysis of anti-CD19 CAR+/- CD19+ cells, anti-CD19 CAR-T cells, CD4/CD8 distribution and activation of T cells in two additional patients. Shown are a PMBCL patient treated with Axicabtagene ciloleucel **(A)** and a transformed Follicular Lymphoma patient treated with Tisagenlecleucel **(B)** 6 and 7 days after treatment, respectively, compared to day 0 before administration of CAR-T cells. Gating strategy was performed according to the **Figures 4**–**6**.

Finally, we measured two additional patients suffering from different B-cell lymphomas (PMBCL, transformed Follicular Lymphoma) and who were treated with either Axicabtagene ciloleucel or Tisagenlecleucel after 6 and 7 days, respectively. The key results of the residual B cell, CAR-T1 and TCR panels are presented in **Figure 9**. As the control for these analyses, we used the patient’s own cells at day 0 under lymphodepleted conditions before administration of the CAR-T cells. Overall, most data are similar to the above shown results. We could not detect any CD19+ B cells nor anti-CD19 CAR+ CD19+ cells in the samples after treatment (**Figures 9A, B**). Among the CD3+ T cells 29.41% (**Figure 9A**) and 14.76% (**Figure 9B**) of the population were positive for the anti-CD19 CAR receptor after 6 and 7 days, respectively. Furthermore, the T cell activation assessed by HLA-DR expression was much higher after 6 and 7 days compared to the patient cells before CAR-T administration at day 0 (**Figures 9A, B**).

In summary, we have developed antibody panels to deeply characterize patient B, T and CAR-T cells using advanced flow cytometry panels and proposed corresponding gating strategies. However, the assays are not limited to these immune cell populations and can be readily expanded to other immune cells important for cancer immunotherapy. As an example, we have also developed antibody panels to phenotype natural killer (NK) cells in the patient blood (see **Supplementary Figures S1** and **S2**). These panels, which assess NK cells through CD56 and CD16 staining, analyze the NK cell status with regards to activating receptors (e.g. NKG2D), cytotoxicity receptors (e.g. NKp46), checkpoint and checkpoint ligand molecules as well as many other the immune modulating molecules. These panels can also be useful for upcoming CAR-NK cell therapies.

## Discussion

Immunotherapy using cancer-directed immune cells is one of the biggest innovations in modern cancer therapy. However, there are still many aspects of CAR-T cell immunotherapy that need to be refined to ensure its lasting success. Indeed, CAR-T cells are genetically modified, living drugs that directly intervene in the patient’s immune system. Therefore, monitoring the immune system of a patient after receiving CAR-T cell infusion might provide important additional information for evaluating the efficacy and safety of the therapy. Moreover, because CAR-T therapy is a novel immunotherapeutic approach, monitoring the patient’s immune system is as crucial for understanding the long-term effects of the overall concept. To monitor anti-CD19 CAR-T cell content *in vivo*, polymerase chain reaction (PCR) is widely used and PCR-results have been reported to correlate with CAR surface expression measured by flow cytometry (4, 20–26). However, flow cytometry allows the identification and characterization of CAR-T cell subpopulations and of patient’s immune cells in a fast way and at a single-cell level. In addition, flow cytometry detects the CAR at the proteomic level and thus can provide information regarding CAR cell functionality (27). Therefore, flow cytometry together with upcoming next-generation sequencing (NGS) approaches (18, 28) enables the comprehensive monitoring of CAR-T cell therapy (29). However, in flow cytometry inter-operator and inter-assay variations can make it difficult to compare data recorded at different times or by different laboratories. Standardization of flow cytometry methods can help to overcome these problems and increase the comparability and reproducibility of acquired data (16). Here we introduced 13 colour/15 parameter flow cytometry assays for standardized research on the fate and immunological impact of CAR-T cells post-infusion.

We characterized CAR-T cells, T cells, B cells and NK cells in the peripheral blood of three patients after the infusion of anti-CD19 CAR-T cells. As expected, the CAR treated patients had only very few leukocytes due to previous chemotherapies and lymphodepletion. We were able to detect the expected amounts of CD19+ B cells in the healthy donor and could not find any residual CD19+ B cells in the anti-CD19 CAR treated patients post-infusion, implicating efficacy of the CAR-T cells. Further, we included the CAR detection in the residual B cell panel to identify potential CAR-positive B cells. In very rare cases it has been described that during the production of the anti-CD19 CAR-T cells residual B cells are also transduced with the anti-CD19 CAR, which could then bind and mask the cell’s own CD19 and thereby escape the therapy leading to death of the patient (30). While PCR methods would miss such CAR-transduced B cells, the flow cytometry panel described here could detect them. Furthermore, we observed that the CD4/CD8 T cell ratio in the CAR patients was lower than in the healthy donor, whereas the activation of T cells (as measured by HLA-DR) was clearly higher in the CAR patients. Fourteen to thirty-five percent of the overall patients T cells were anti CD19 CAR-T cells with varying CD4/CD8 ratios between the patients (approx. 0.3 – 1.8). In addition, our flow cytometry assays were designed to measure a large repertoire of T cell fitness parameters, which included exhaustion markers and checkpoint as well as other immunomodulating molecules. We also observed that the DLBCL patient’s CAR-negative T cells showed a very high expression of the exhaustion marker CD57, whereas the CAR-positive T cells were low in CD57, which needs to be confirmed in a larger sample cohort. Regarding the expression of checkpoint and immunomodulating markers, we detected a general trend toward higher expression in the patient T cells than in the healthy donor T cells. With the CAR-T3 panel we were able to analyze the cells’ cytotoxic potential by measuring intracellular perforin, granzyme B, IL-2, TNF-α and IFN-γ. We found that, in contrast to the healthy donor, the CAR patient’s T cells are loaded with perforin and granzyme B, both unstimulated and stimulated, indicating their cytotoxic potential. Furthermore, the T cells of the healthy donor and the CAR treated patient produced IFN-γ after stimulation indicating an activity of the cells.

The goal of this work was to introduce standardized flow cytometry research assays for the study of infused anti-CD19 CAR-T cells including the interacting immune system. Therefore, we designed comprehensive antibody panels and provided detailed gating strategies for all panels using the peripheral blood of a DLBCL patient as an example (**Figures 3** **–** **8**). In addition, to show the strength and versatility of the assays, we have measured two more patients with different B cell lymphomas (PMBCL, Follicular Lymphoma), and which were treated with different CAR-T cell products (Axicabtagene ciloleucel or Tisagenlecleucel, **Figure 9**). We acknowledge that our study has several limitations. First of all, the obtained results only represent a snap-shot of one healthy and a very small number of patient donors. For instance, it is well known that the presence and the status of CAR-T cells in the peripheral blood highly vary over time (7). From a technological point of view, it is important to note that the CAR patients had very low leukocytes numbers, which might skew the staining procedure and the comparison to a healthy donor with comparatively higher leukocytes numbers. The low number of leukocytes in the lymphodepleted CAR patients also determines the lower limit of detection (LLOD), which with the achieved number of leukocytes is 1%). Furthermore, here we analyzed B, T and NK cells but other immune cell populations might be of interest to obtain a comprehensive picture of the patient immune status. Taken together, although our data visualize the suitability of the introduced advanced flow cytometry assays, further experiments with more donors and time points are needed to verify the results and to subsequently optimize the antibody panels. Such future work may best be performed in multi-center studies.

In summary, we have introduced flow cytometry research assays for studying anti-CD19 CAR-T cells and the interacting immune system *in vivo*. The underlying principle using pre-formulated, single tube antibody cocktails, makes these assays a promising method for the standardized monitoring of CAR-T immunotherapy and therefore has also been suggested by others (31, 32). The DURAclone technology used here has been successfully applied for the detection of regulatory T cells (Tregs), naïve/memory T cells and other immune cells in the peripheral blood of healthy human donors in previous work (33–35). Furthermore, the feasibility of this technology for immuno-monitoring of cancer patients has been proven by the detection of minimal residual disease (MRD) in myeloma patients and by analyzing blood cells of cancer patients in response to immunotherapeutic nanoparticles (36, 37). In addition, the introduced technology is not limited to CD19-targeting CAR approaches and can be readily adapted to the evaluation of other CAR immunotherapies. In this regard, the design of the CAR-T 1-3 panels and the RE ALB panel provides a well standardized framework, which allows for drop-in of any specific anti-CAR probes (e.g. soluble CAR-targeted proteins, anti-idiotypic antibodies) labeled with the bright and well-established PE dye. Indeed, it is expected that several new CAR-T cell products will reach market authorization soon. Moreover, the number of upcoming future clinical trials investigating CAR-T cells and other CAR-engineered immune effector cells such as NK cells or macrophages continues to expand (6, 38–40). However, the potential increase in CAR therapies exceeds the current manufacturing capacity and only a fraction of patients who could benefit from CAR-T therapy are currently receiving it (41). Therefore, the *Good Manufacturing Practice (*GMP)-compatible production of CAR-T cells requires continuous process improvement to keep up with the fast-growing medical demand (42). In this regard, first semi-automated cell processing systems have been successfully used for the GMP-compatible, clinical-grade production of CAR-T and CAR-NK cells (7, 43–48). Monitoring CAR-T cell production from the starting leukapheresis material over to the process of production/upscaling to the final product release requires standardized quality control (QC) tools. Thus, we envision that our flow cytometry assays can also serve as novel QC tools during CAR-T cell production, in particular when linked to automated data analysis and machine learning technologies, which is within reach as previously shown (49, 50).

## Data Availability Statement

The raw data supporting the conclusions of this article will be made available by the authors, without undue reservation.

## Ethics Statement

The studies involving human participants were reviewed and approved by Ethical Committee at the Medical Faculty, Leipzig University Käthe-Kollwitzstraße 82 04109 Leipzig. The patients/participants provided their written informed consent to participate in this study.

## Author Contributions

UB, UP, VV, TM, MB, SF, US, and UK designed the research. RW, AB, and AP ran the experiments. UB, RW, AB, MK, ARB, AQ, AP, VV, UP, US, SF, and UK analyzed results. UB, RW, and AB created the figures. UB, RW, AB, US, SF, and UK wrote the paper. All authors contributed to the article and approved the submitted version.

## Funding

This study was supported by the Fraunhofer Society, the Fraunhofer Cluster of Immune Mediated Diseases (CIMD), and the Leistungs- und Transferzentrum Chemie- und Biosystemtechnik which is supported by Sächsische Aufbaubank (SAB). This work was co-financed with tax revenues on the basis of the budget approved by the members of the Saxon state parliament. In addition, this project has received funding partly from the Innovative Medicines Initiative 2 Joint Undertaking (JU) under grant agreement No 853988. The JU receives support from the European Union’s Horizon 2020 research and innovation programme and EFPIA and JDRF INTERNATIONAL.

## Conflict of Interest

TM, MB, and MK are employees of Beckman Coulter Life Sciences.

The remaining authors declare that the research was conducted in the absence of any commercial or financial relationships that could be construed as a potential conflict of interest.

## Supplementary Material

Supplementary Figure 1 Characterization of NK cells in the peripheral blood of a healthy individual and a patient treated with anti-CD19 CAR-T cells by the panel NK1. Flow cytometric gating strategy (illustrated by arrows) and phenotyping of NK cells in a patient 9 days after CAR-T cell reinfusion **(A–L)** in comparison to a healthy donor **(a–l)**: After lymphocyte gating **(a, A)** and exclusion of doublets **(b, B)**, as well as T cells and monocytes gating by their expression of CD3 and CD14 **(c, C)**, respectively, NK cells were divided in CD16+/CD56bright and CD16+/CD56dim cells **(d, D)**. CD56bright and dim cells **(d, D)** in both healthy control **(d–l)** and in the CAR patient **(D–L)** were analyzed for the expression of: Activating receptor NKG2D and natural cytotoxicity receptor NKp46 **(e, E)**, checkpoint ligand PD-L1/CD274 **(f, F)**, checkpoints CD137 and PD-1 **(f, F, g, G, j, J)**, the immune modulating molecules TIGIT, CD155, CD226, **(h, H, j–l, J–L)** and the activation marker CD25 **(i, I)**.

Supplementary Figure 2 Characterization of NK cells in the peripheral blood of a healthy individual and a patient treated with anti-CD19 CAR-T cells by the panel NK2: Flow cytometric gating strategy (illustrated by arrows) and phenotyping of NK cells in a patient 9 days after CAR-T cell reinfusion **(A–L)** in comparison to a healthy donor **(a–l)**: After lymphocyte gating **(a, A)** and exclusion of doublets **(b, B)**, as well as T cells and monocytes gating by their expression of CD3 and CD14 **(c, C)**, respectively, NK cells were divided in CD16+/CD56bright and CD16+/CD56dim cells **(d, D)**. CD56bright and dim cells in both healthy control **(d–l)** and in the anti CD19 CAR patient **(D–L)** were analyzed for the expression of: Activating receptor NKG2D and natural cytotoxicity receptor NKp46 **(e, E)**, the activation markers HLA-DR **(fF)** and CD69 **(g, G)** as well as checkpoints CD278 (ICOS) **(h, H)**, LAG-3 **(i, I)**, CD134 (OX40) **(j, J)**, TIM-3 **(k, K)** and VISTA **(l, L)**.
