# Supplementary figures and images for: Advanced Flow Cytometry Assays for Immune Monitoring of CAR-T Cell Applications
Source: Front Immunol. 2021 May 3;12:658314. doi: 10.3389/fimmu.2021.658314 (PMC8127837; doi:10.3389/fimmu.2021.658314)

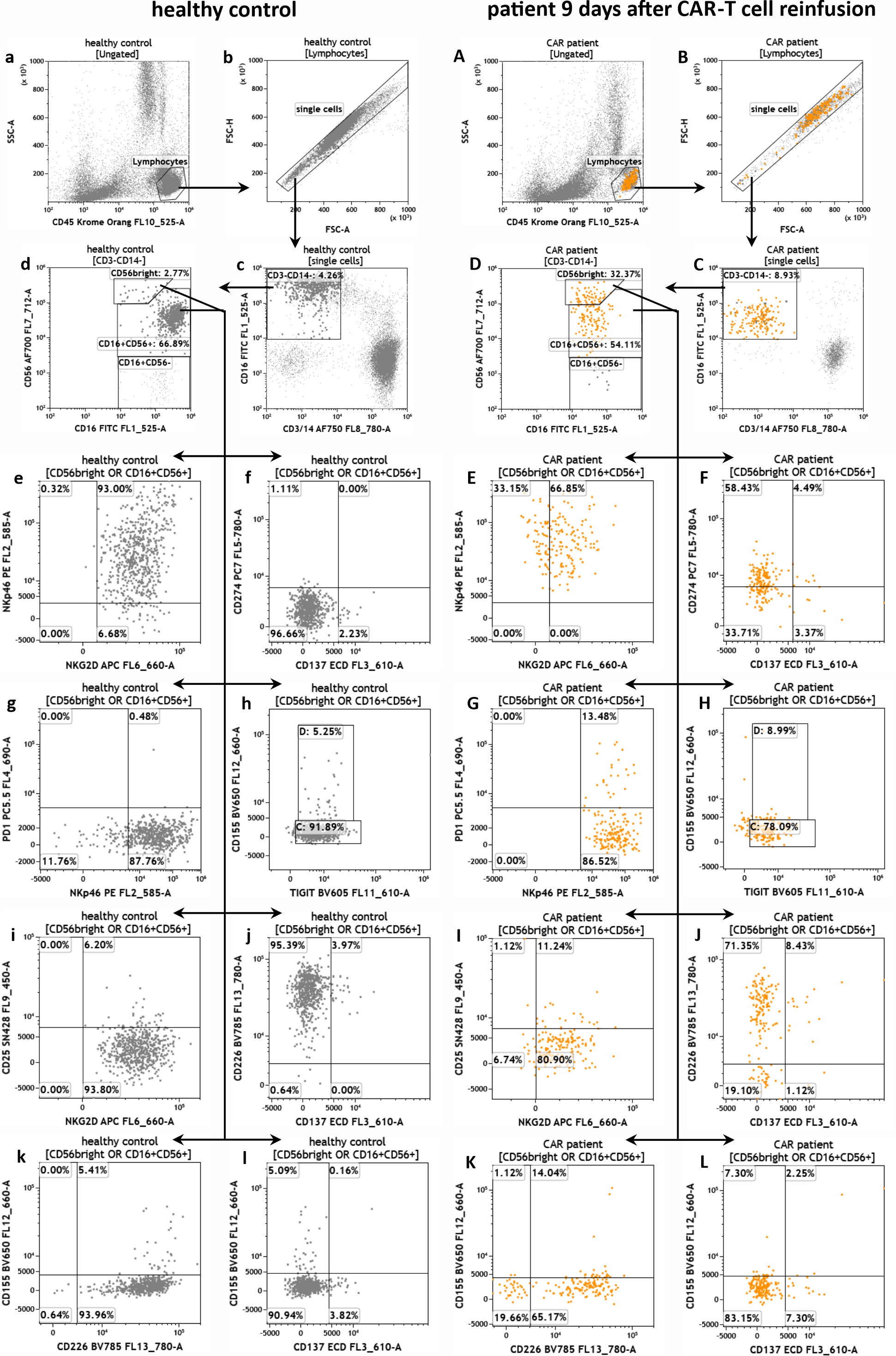

Supplement: Supplementary Figure 1 — Characterization of NK cells in the peripheral blood of a healthy individual and a patient treated with anti-CD19 CAR-T cells by the panel NK1. Flow cytometric gating strategy (illustrated by arrows) and phenotyping of NK cells in a patient 9 days after CAR-T cell reinfusion (A–L) in comparison to a healthy donor (a–l): After lymphocyte gating (a, A) and exclusion of doublets (b, B), as well as T cells and monocytes gating by their expression of CD3 and CD14 (c, C), respectively, NK cells were divided in CD16+/CD56bright and CD16+/CD56dim cells (d, D). CD56bright and dim cells (d, D) in both healthy control (d–l) and in the CAR patient (D–L) were analyzed for the expression of: Activating receptor NKG2D and natural cytotoxicity receptor NKp46 (e, E), checkpoint ligand PD-L1/CD274 (f, F), checkpoints CD137 and PD-1 (f, F, g, G, j, J), the immune modulating molecules TIGIT, CD155, CD226, (h, H, j–l, J–L) and the activation marker CD25 (i, I). [file Image_1.jpeg]

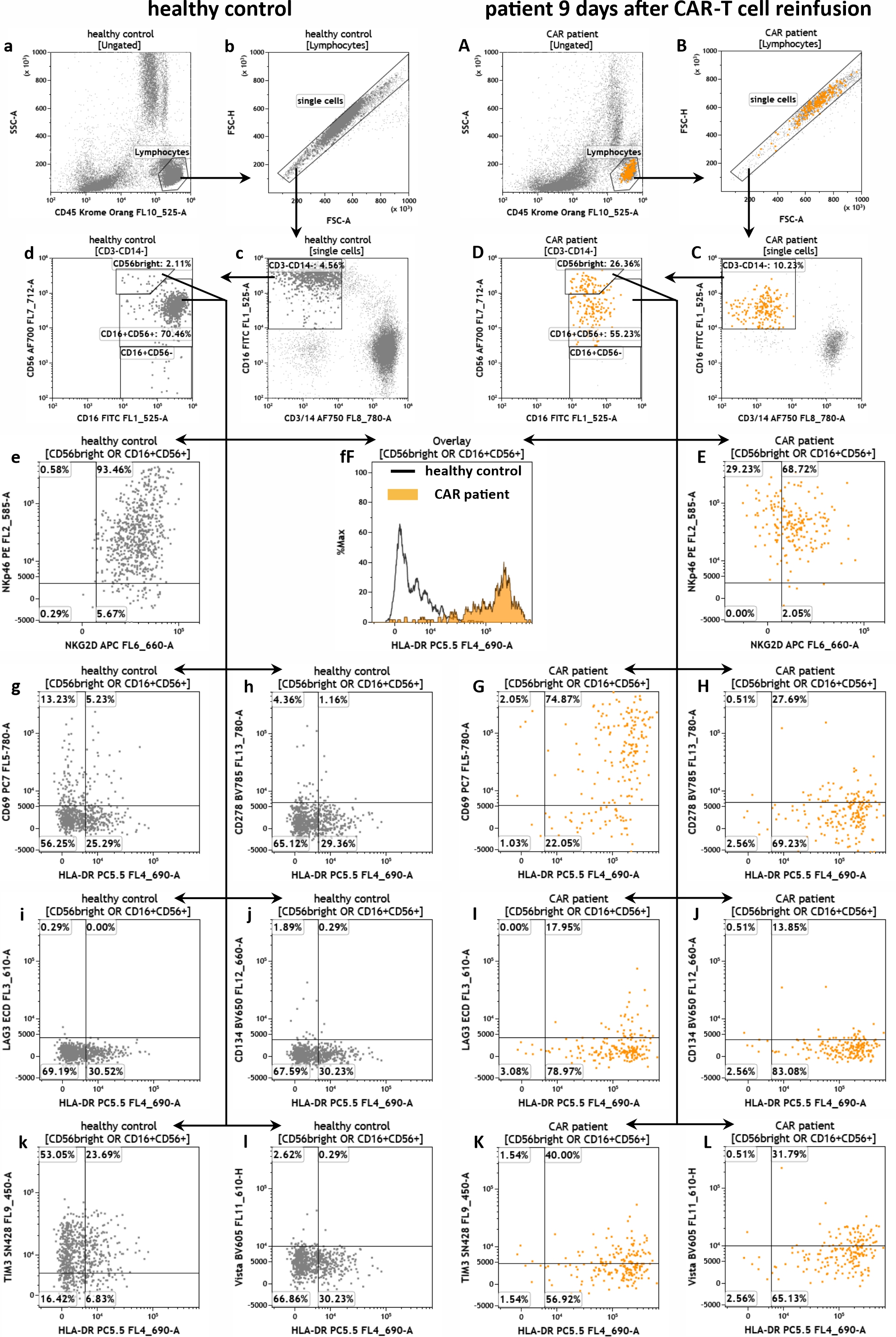

Supplement: Supplementary Figure 2 — Characterization of NK cells in the peripheral blood of a healthy individual and a patient treated with anti-CD19 CAR-T cells by the panel NK2: Flow cytometric gating strategy (illustrated by arrows) and phenotyping of NK cells in a patient 9 days after CAR-T cell reinfusion (A–L) in comparison to a healthy donor (a–l): After lymphocyte gating (a, A) and exclusion of doublets (b, B), as well as T cells and monocytes gating by their expression of CD3 and CD14 (c, C), respectively, NK cells were divided in CD16+/CD56bright and CD16+/CD56dim cells (d, D). CD56bright and dim cells in both healthy control (d–l) and in the anti CD19 CAR patient (D–L) were analyzed for the expression of: Activating receptor NKG2D and natural cytotoxicity receptor NKp46 (e, E), the activation markers HLA-DR (fF) and CD69 (g, G) as well as checkpoints CD278 (ICOS) (h, H), LAG-3 (i, I), CD134 (OX40) (j, J), TIM-3 (k, K) and VISTA (l, L). [file Image_2.jpg]
